# Characterization of CLA-producing *Butyrivibrio* spp. reveals strain-specific variations

**DOI:** 10.1007/s13205-016-0401-2

**Published:** 2016-03-10

**Authors:** S. K. Asraf Hussain, Anima Srivastava, Ashish Tyagi, Umesh Kumar Shandilya, Ashwani Kumar, Sachin Kumar, Surbhi Panwar, Amrish Kumar Tyagi

**Affiliations:** 1 Dairy Cattle Nutrition Division, National Dairy Research Institute, Karnal, 132001 Haryana India; 2 Seth Jai Parkash Mukand Lal Institute of Engineering and Technology (JMIT), Radaur, 135133 Haryana India; 3 Department of Nutrition Biology, Central University of Haryana, Mahendergarh, 123029 Haryana India

**Keywords:** *Butyrivibrio fibrisolvens*, Conjugated linoleic acid, Linoleic acid, Biohydrogenation, Ruminants

## Abstract

Conjugated Linoleic Acid (CLA), a fatty acid with high nutraceutical value is produced in rumen by resident bacterial species, especially *Butyrivibrio spp*. The present study was undertaken to examine the diversity of indigenous *Butyrivibrio spp*. from rumen liquor of Indian ruminants. The isolates were screened for their CLA production capability at different level of linoleic acid (LA) (0, 200, 400, 600, 800 μg/ml) at different time intervals (0, 2, 4, 6, 12, and 24 h). A total of more than 300 anaerobic cultures were isolated and 31 of them were identified as *Butyrivibrio* spp. based on morphological, biochemical and molecular characterization. Further, molecular characterization revealed that a large portion (67.7 %) of isolated *Butyrivibrio* belonged to *Butyrivibrio fibrisolvens* (*B. fibrisolvens*) species which is considered to be the most active bacteria amongst the rumen bacteria populace in terms of CLA production. Bacterial isolate VIII (strain 4a) showed highest CLA production ability (140.77 μg/ml) when incubated at 200 μg/ml LA for 2 h, which is 240 % higher than the isolate XXVII, *Butyrivibrio proteoclasticus* (B. *proteoclasticus*) showing lowest CLA production (57.28 μg/ml) amongst the screened isolates. It was evident from the observations recorded during the course of experiments that CLA production ability is strain specific and thus did not follow a single pattern. CLA production also varied with time of incubation and concentration of free linoleic acid supplemented in the growth medium. The results of these findings put forward a strain that is high CLA producer and can be further exploited as an additive for enhancing meat and milk quality in ruminants.

## Introduction

CLA is a mixture of positional and geometric isomers of linoleic acid (cis-9, cis-12, C_18:2_) with two conjugated double bonds at various carbon positions in the fatty acid chain. It is formed as an intermediate during the biohydrogenation of LA by linoleic acid isomerase from the rumen bacteria (Li et al. 2012).

The increased interest in its anti-cancerous, anti-atherogenic, immune-modulatory properties (Hino et al. 2012; Chinnadurai et al. 2013; Dilzer et al. 2015) has provided an impetus to the research, in past decade, leading to better understanding of underlying mechanisms and its natural incorporation in the food products pertaining to its high nutraceutical value. It has now been understood that rumen can be a site of therapeutic interference for enhancement of natural CLA content and rumen microorganisms are major influential factors in the rumen fatty acid biohydrogenation process and ultimately formation of CLA.

Rumen bacteria responsible for biohydrogenation of fatty acids are categorized into two distinct groups (Type A and B), depending on their ability to hydrogenate both the dienoic and the monoenoic acids or only the monoenoic acid. Type A bacteria convert the LA to CLA, i.e. *Butyrivibrio*, *Micrococcus*, *Ruminococcus*, *Lactobacillus* and dominated by *B. fibrisolvens* which has higher CLA-producing capacity than other ruminal bacteria. Type B bacteria are producing stearate from PUFA in rumen, i.e. *Fusocillus*, *B. proteoclasticus* (Ha et al. 1989).

Several species of CLA-producing bacteria have been isolated from the rumen, intestine and starter cultures used in the dairy industry (Kepler et al. 1966; Willems et al. 1986; Griinari and Bauman 1999; Lin et al. 1999; Kritchevsky 2000; Coakley et al. 2003; Kim 2003; Rosberg-Cody et al. 2004). According to these reports, the prevalent ruminal bacterium *B. fibrisolvens* isomerizes LA to CLA faster than any other bacterial species. To date most studies have analysed and explained the effects of *B. fibrisolvens* on meat and milk products, several others have demonstrated its healthy effects in mice (Ohkawara et al. 2006; Hino et al. 2012; Chinnadurai et al. 2013). In this study we have extended the knowledge of *Butyrivibrio* diversity, particularly in rumen of ruminant population of Indian origin, by analysing the 16SrRNA sequencing data. The strains of *Butyrivibrio spp* were found to exhibit large diversity and *B. fibrisolvens* was present in large number amongst other species (67.7 %).

Apart from the diversity study we have also demonstrated that ability of a rumen microbe to use LA as a precursor for CLA production is highly strain specific. This also suggests that using feed with high LA content as prebiotic and a high CLA-producing bacterium along with it can have a synergistic effect resulting in ruminant food product with high nutraceutical content.

## Materials and methods

### Sample collection and isolation of *Butyrivibrio*

The rumen liquor was collected in thermos flask from fistulated buffalo, cattle and goat maintained at cattle yard, NDRI, Karnal, while that of sheep was collected from slaughter house of Karnal. After collection, it was centrifuged at 2000×*g* for 10 min to remove protozoa and fungi. M704 media was prepared as described by the Deutsche Sammlung von Mikroorganismen and Zellkulturen (DMSZ 1993). The diluted rumen liquor, preferably 10^5^–10^6^ dilutions, was inoculated on plates containing DSMZ 704 media. The composition of media was as follows: Peptone, 0.2 %; Yeast extract, 0.2 %; Starch Soluble, 0.1 %; K_2_HPO_4_, 0.06 %; VFA, 10 mL/L (Acetic acid, 6.85 mL; Propionic acid, 3 mL; Butyric acid, 1.85 mL; 2-Methyl butyric acid, 0.55 mL; Isobutyric acid, 0.5 mL; Valeric acid, 0.55 mL; 0.2 M NaOH, 700 mL; adjust pH to 7.5 with 0.2 M NaOH); Glycerol, 0.05 %; Rumen Fluid, 150 mL/L; Haemin 1 mg/L, Mineral Solution, 75 mL/L (KH_2_PO_4_, 6 g; (NH_4_)_2_SO_4_, 6 g; NaCl, 12 g; MgSO_4_.7H_2_O, 2.55 g; CaCl_2_. 2H_2_O, 1.9 g per litre); resazurin. Two percent of reducing agent containing 1.25 % of each L-cystine HCl and Na_2_S. 9H_2_O and 2 % of sugar solution (5 % each of glucose, maltose and cellobiose) were added in media. The media was prepared anaerobically using gassing manifold and the plates were incubated at 39 °C for 48 h inside the anaerobic chambers.

### Phenotypic characterization of isolates

The isolates were preserved as pure cultures. Streaking was done on DSMZ 704 plates prepared anaerobically. Single colony was used to inoculate the broth and grown for 16–18 h. The same culture was used for Gram staining to check the purity.

Preliminary identification was done on the basis of Gram staining and several biochemical tests including various carbohydrate-fermentation tests (Table 1).

**Table 1:** Common phenotypic characteristics of *Butyrivibrio* isolates

Tests	Response	Carbohydrate-fermentation tests			
		Name of sugars	Response	Name of sugars	Response
Motility	+	Inulin	–	Mannose	–
Gas production	+	Sucrose	+	Inositol	+
Catalase	–	Fructose	+	Cellobiose	–
Methyl red	–	Trehalose	+	Arabinose	–
Vogues Prauskauer	–	Dextrose	+	Galactose	+
Indole test	–	Maltose	+	Mannitol	+
Nitrate reduction	+	Xylose	+	Lactose	+
Gelatin liquefaction	–				
Oxidation fermentation	+				
Metabolites test	+				

^#^pH of the isolates during sugar fermentation tests were in between 6.2 and 7

### PCR identification

#### Primer designing

PCR primers were designed from *Butyrivibrio* spp. 16SrRNA gene sequence retrieved from NCBI database of *Butyrivibrio* spp. strains with the help of DNA star/CLCBio6 software. Briefly, the primers were designed by finding conserved region in 16SrRNA sequences of concerned species through ClustalW in DNA Star software itself. The identified sequences were then used with Primerselect option in the same software. Four pairs of primers namely, ButUnivF/ButUnivR1 and ButUnivF/ButUnivR2 (*Butyrivibrio* genus-specific universal primers), BfibF/BfibR (*B. fibrisolvens* species-specific primers) and ButHungF/ButHugnR (*Butyrivibrio hungatei* species-specific primers), ButProF/ButProR (*B. proteoclasticum* species-specific primers) based on 16S rRNA sequence of *Butyrivibrio* strains available on NCBI were designed. The detailed description of these primers along with expected amplified products is given Table 2.

**Table 2:** Genus- and species-specific primers of *Butyrivibrio*

Primer region	Species and Primer sequence	Product size (bp)	Annealing temp (°C)
16s rRNA	**ButUnivF**: 5′-CTATCAGCAGGGAAGAAAG-3′ **ButUnivR1**: 5′-GTTAGCGACGGCACTGA-3′	420	53.5
16s rRNA	**ButUnivF**: 5′-CTATCAGCAGGGAAGAAAG-3′ **ButUnivR2**: 5′-CCGTCAATTCCTTTGAGTTTC-3′	482	54
16s rRNA	**Species-specific (** ***Butyrivibrio fibriosolvens*** **)** **BfibF:** 5′-ACACACCGCCCGTCACA-3′ **BfibR**: 5′-TCCTTACGGTTGGGTCACAGA-3′	220	55
16s rRNA	**Species-specific (** ***Butyrivibrio proteoclasticus*** **)** **ButproF:** 5′-ACTCCTACGGGAGGCAG-3′ **ButproR:** 5′-CTGAATGCCTATGGCACCCAA-3′	505	52
16s rRNA	**Species-specific (** ***Butyrivibrio hungatei*** **)** **ButHungF**: 5′-CCGCATAAAACAGCAGAGTCGCAT-3′ **ButHungR:** 5′-TAGCACGTGTAGCCCAAG-3′	1150	58

#### PCR-based identification

The genomic DNA was isolated from the bacterial cultures using Bacterial DNA isolation Kit (Qiagen, USA) by following manufacturer’s instructions. Reaction mixture (25 µL) for PCR amplification contained DNA, 1X Dream Taq (Fermantas, USA), primers (10 µM) and nuclease-free water to make up the final volume. Amplifications were performed in a Quanta Biotech thermocycler with an initial denaturation step of 94 °C for 3 min followed by 30 cycles of 94 °C for 30 s, 52–58 °C (according to primer) for 45 s and 72 °C for 30 s and a final extension of 72 °C for 10 min. The resultant PCR products were sequenced (Chromus Biotech, India). Sequence percent identity was analysed by BLAST within GenBank and finally submitted to NCBI.

#### Sequence analyses

Comparison of sequences was performed using BLAST program (Altschul et al. 1990) to the reported nucleotide sequences in GenBank database accessible through NCBI (National Centre for Biotechnology Information—http://www.ncbi.nlm.nih.gov). Sequences having 98 % and above similarity with *B. fibrisolvens* (*B. fibrisolvens* Bryant and Small (ATCC^®^ 19171™, Acc No. U41172.1) were retrieved and used further for analysis. The sequences were edited with BioEdit v5.0.9 (Hall 1999). The sequences were aligned with Multiple Sequence Comparison by Log-Expectation (MUSCLE) and the phylogeny was ascertained using PhyML with aLRT (approximate Likelihood-Ratio Test) or bootstrap analysis for branches. The tree was rendered using TreeDyn. The numbers next to each node, in red, represent a measure of support for the node, where 1 represents maximal support. These have been computed by bootstrapping analyses.

### Determination of CLA production potential: spectrophotometric method

The isolated pure anaerobic cultures of *Butyrivibrio* spp. were supplemented with different concentrations of LA (50, 100, 150, 200, 250, 400, 600, 800 µg/ml of broth). Stock solution of LA (99 % purity) was prepared in 1 % aqueous solution of Tween 80. The medium was inoculated with 1 % bacterial culture and the tubes were incubated at 39 °C in anaerobic chamber. After incubation at different time period tubes were placed in an ice-water bath for arresting the bacterial activity. A rapid screening method described by Barrett et al. (2007) was used for analysing the CLA-producing capability of bacterial strains. In brief, the method employed in this study involved the use of a UV-transparent and colourless 96-well plate to detect total CLA production by bacterial cultures at a wavelength of 233 nm. To verify the suitability of this method, a standard curve was constructed for the absorbance at 233 nm. The graph demonstrated that an increase in the CLA concentration (from 0.531 to 65.935 µg/ml) coincided with a linear increase (*R* ^2^ = 0.9947) in absorbance for total CLA. The CLA concentrations in culture supernatants with an absorbance at 233 nm could be calculated from the linear trend line of the standard curve using the equation *y* = 0.0984*x* + 0.147. This method was used for primary screening for CLA production, further the same was confirmed by gas chromatography.

### Determination of CLA production potential: gas chromatography method

Fat was extracted from media by the method of Ha et al. (1989). The extracted fat was hydrolyzed with 1 ml of 1 N methanolic sodium hydroxide in a boiling water bath for 15 min and then cooled to room temperature for 5 min. 1 ml of 2 N hydrochloric acid and 2 ml of chloroform were added to the tube containing methanolic sodium hydroxide and vortexed for 4 min, followed by centrifugation for 10 min at 2200 rpm. The organic layer (lower layer) was collected and evaporated to dryness under vacuum or under the steam of nitrogen. A stock solution of CLA (1 mg/ml) in acetonitrile was prepared. A working standard solution was prepared by adding (500 μl) stock solution to 2 ml of acetonitrile and it gives 4 μg of CLA in 20 μl of standard to be injected.

**Fig. 1:**
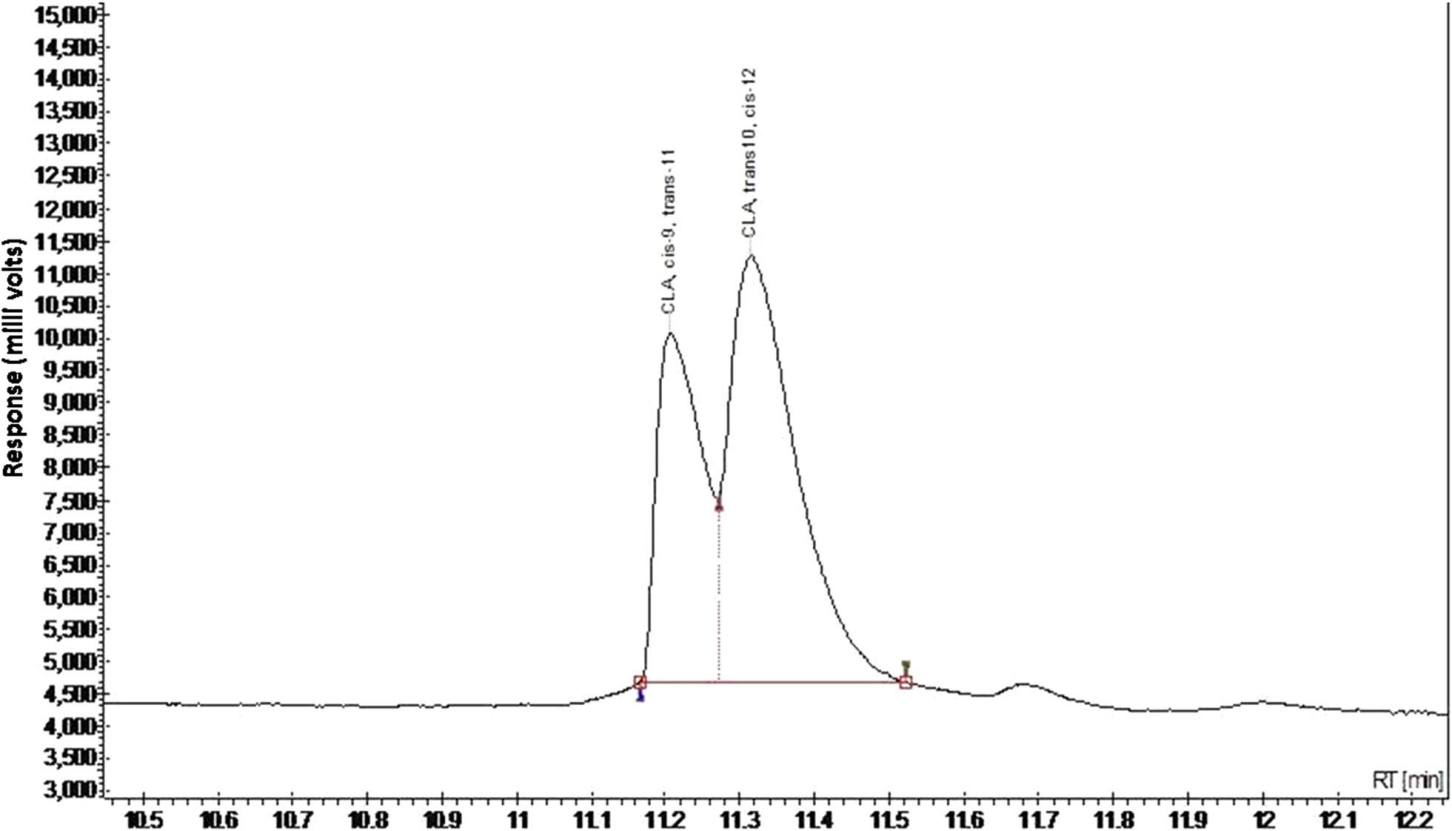
GC chromatogram of isomers of CLA (Standard)

Methyl esters were separated using a GC (450-GC, Bruker, USA) equipped with an SGE Forte GC capillary column (60 m × 0.25 mm × 70 μm-BPX70). Helium was used as carrier gas at constant inlet pressure (205 kPa). The injector and detector temperature were 260 and 270 °C, respectively, and the split ratio was 1:10. The initial oven temperature was 120 °C and increased by 2 °C/min to 240 °C for 55 min. The identification of individual fatty acid was based on a commercial standard mixture (Supelco, Belfonte, USA) and published isomeric profiles (Fig. 1).

Spectrophotometric method was used for primary screening, further the same was confirmed by gas chromatography. The results mentioned in manuscript are obtained by GC.

## Results

**Fig. 2:**
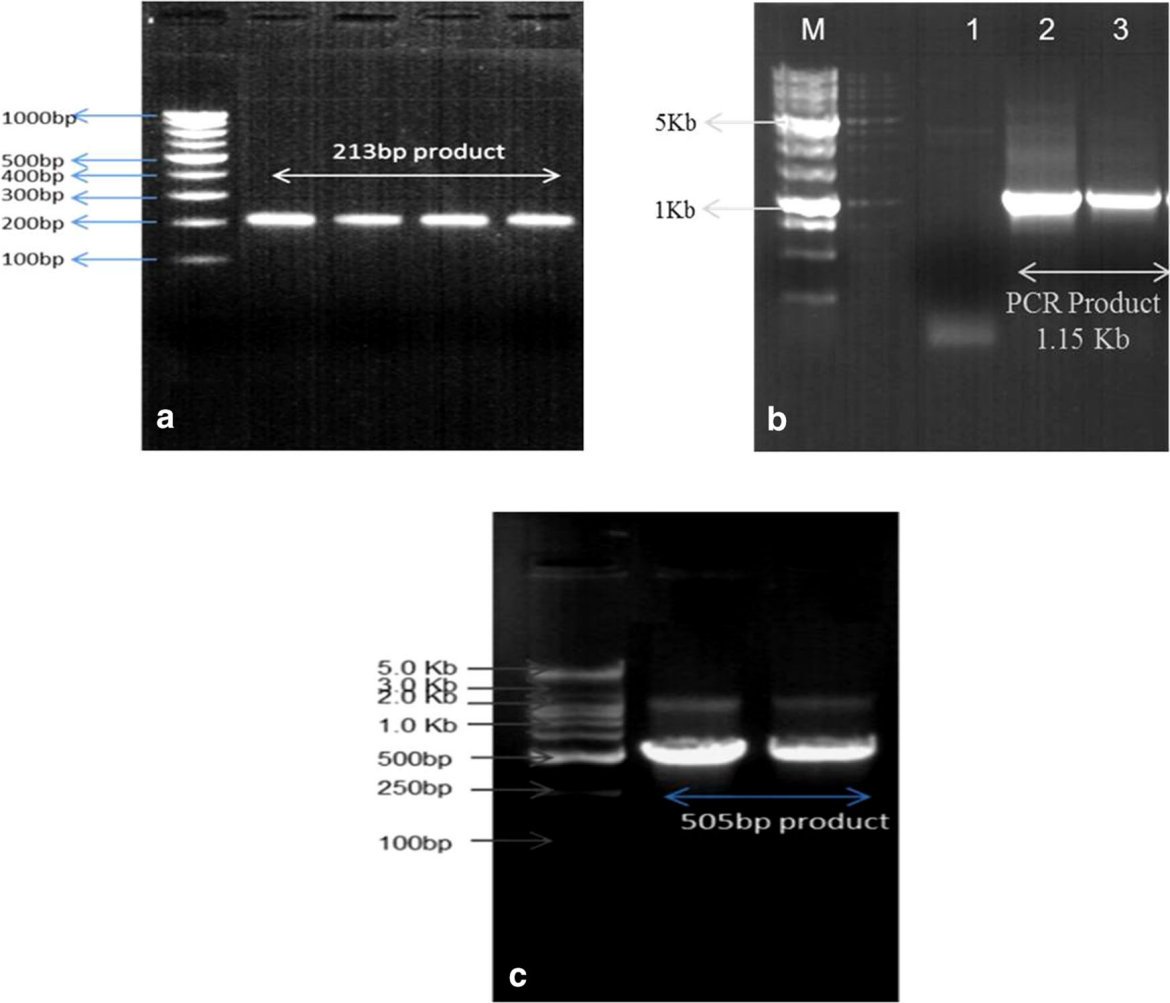
Species level identification of the bacterial isolates confirmed as *Butyrivibrio* spp. **a** Bands of genomic DNA of *B. fibrisolvens* using primer pairs BfiBF/BfiBR on gel; **b** Bands of genomic DNA of *B. hungatei* using primer pairs ButHungF/ButHungR on gel; **c** Bands of genomic DNA of *B. proteoclasticus* using primer pairs ButproF/ButproR on gel

A total of 300 pure anaerobic bacterial isolates were picked on the basis of colony morphology for further characterization. Prefatory characterization of isolates was done by Gram’s staining as well as with biochemical tests and the results were matched with “Bergey’s Manual of Determinative Bacteriology” (Holt et al. 1994), which primarily approved the isolates as *Butyrivibrio spp*. Furthermore, 31 strains including 21 *B. fibrisolvens*, 4 *B. hungatei* and 6 *B. proteoclasticus* were identified by PCR with genus- and species-specific primers by sequence analysis of PCR products (Table 2; Fig. 2). The results showed that among the isolated *Butyrivibrio*, *B. fibrisolvens* predominated the microflora with 67.7 %, followed by *B. proteoclasticus* (19.35 %) and *B. hungatei* (12.9 %).

The PCR products were further custom sequenced (Chromus Biotech, India) for homology and phylogenetic analysis. BLASTn analysis of the sequences demonstrated high percentage homology of the isolate sequences with *Butyrivibrio* spp. sequences already indexed at NCBI. Sequence analysis using BLASTn revealed that the sequences of 21 isolates showed close similarity (98–99 %) with *B. fibrisolvens* (*B. fibrisolvens* Bryant and Small (ATCC^®^ 19171™, Acc No. U41172.1). Similarly, the sequences of four isolates showed close similarity with *B. hungatei*. Strains 7a, 61, 67, 20, 24, and 50 showed 99 % similarity to *B. proteoclasticus*; strains 33, 38, 47, 63, 65, 37, and 28 showed 98 % similarity to *Pseudobutyrivibrio spp*. Besides this, strains 9A2, 54, and 23 showed close similarities to butyrate-producing bacteria; strains 13, 36, and 2 were observed to show good homology to rumen bacteria; strains 1, 3, 4, 7, and 6 were observed to be *Bacillus dendrites (B. dendrites)*. Strain 5 showed good similarity to *Bacillus pumilus (B. pumilus)*, whereas isolate 3K2 showed close similarity to *Paenibacillus* spp. while strain 12 and 53 showed similarity to *Provedenciavermicola*. BLASTn search of the remaining isolates indicated that they showed good homology with uncultured bacteria such as EU461824.1, KT797946.1 and AM275783.1.

### Heterogeneity analysis

**Fig. 3:**
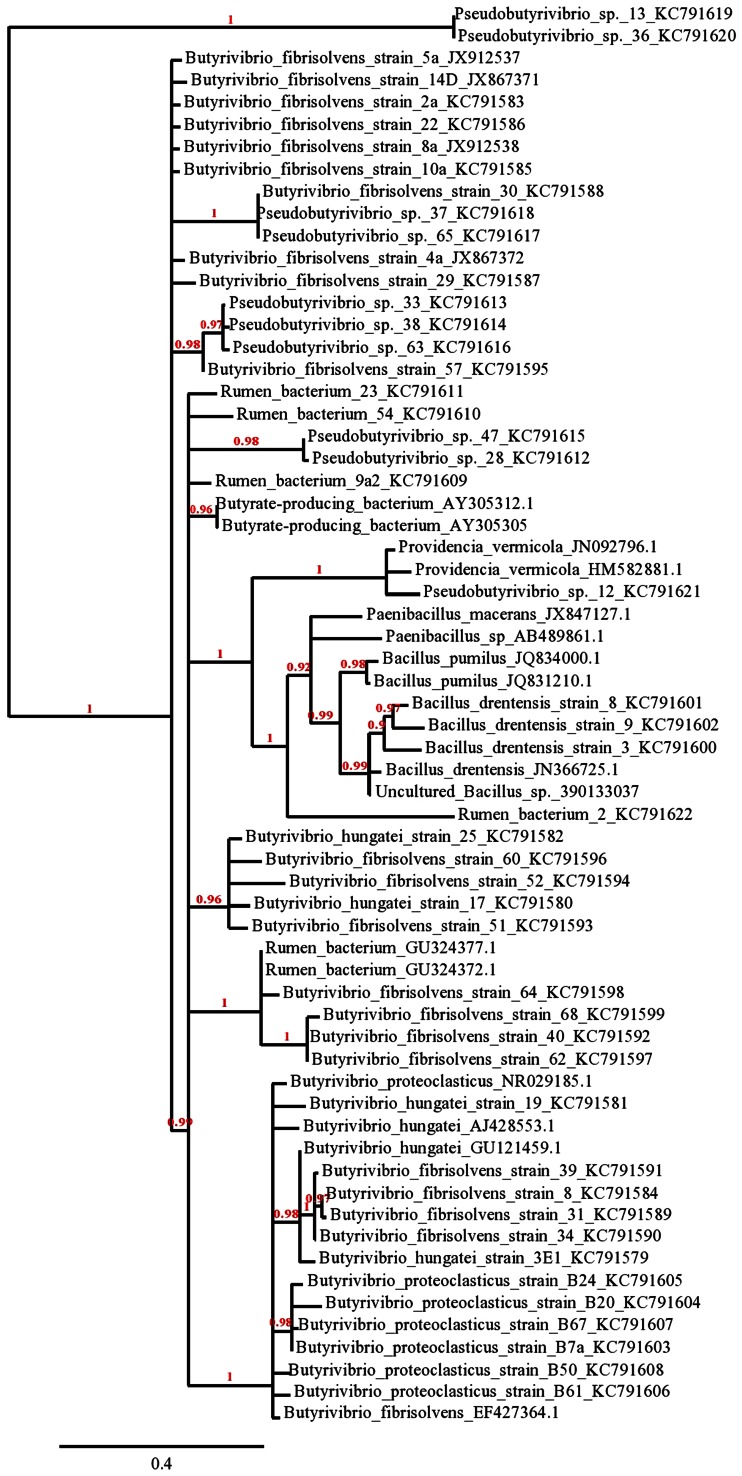
The *numbers* next to each node, in *red*, represent a measure of support for the node, where *1* represents maximal support and confidence. The assessments of “confidence” for each clade of the tree are based on the proportion of bootstrap trees showing that same clade. These proportions are the bootstrap confidence values

A phylogenetic tree was constructed and it was evident that the isolated strains have similar evolutionary lineage. A high bootstrap confidence value shows that the strains are from similar species and are clustered together in the tree. For example, the clade with strains 33, 38, 63 seen in Fig. 3 appeared in 197 of the 200 bootstrap trees, for an estimated confidence value of 0.97. Phylogenetic analysis revealed that strains namely, 2a, 22, 14D, 5a, 8a, 10a, 4a, 31, 34, 39, 8, 68, 40, 62, 64, 51, 3E1 were clustered with high bootstrap value into a group within the *B. fibrisolvens* group, confirming that these strains are *B. fibrisolvens*. Strains 30, 29, 57 closely related (having close similarity to rumen bacteria from BLAST results) to *Pseudobutyrivibrio* clade strains 63, 38, 33. While 37, 65, 28, 47 are almost at similar evolutionary distance from one another. Similarly B50, B67, B24, B20, B61, 7a were observed to be of the same clade.

Interestingly 3E1, 17, 25 strains belonging to *B. hungatei* group are in different clade from strain 19 which is at close distance to *B. fibrisolvens* strains. A non-*Butyrivibrio* clade, named as dendrites clade, contain the strains 8, 9, and 3 (Fig. 3).

Phylogenetic analysis through maximum likelihood and minimum parsimony resulted in the complete identification of these bacteria which resulted in 21 *B. fibrisolvens*, 4 *B. hungatei* and 6 *B. proteoclasticus* strains. The remaining strains were observed to be *Pseudobutyrivibrio* spp. (9) and other non-*Butyrivibrio* groups.

### CLA production potential

**Fig. 4:**
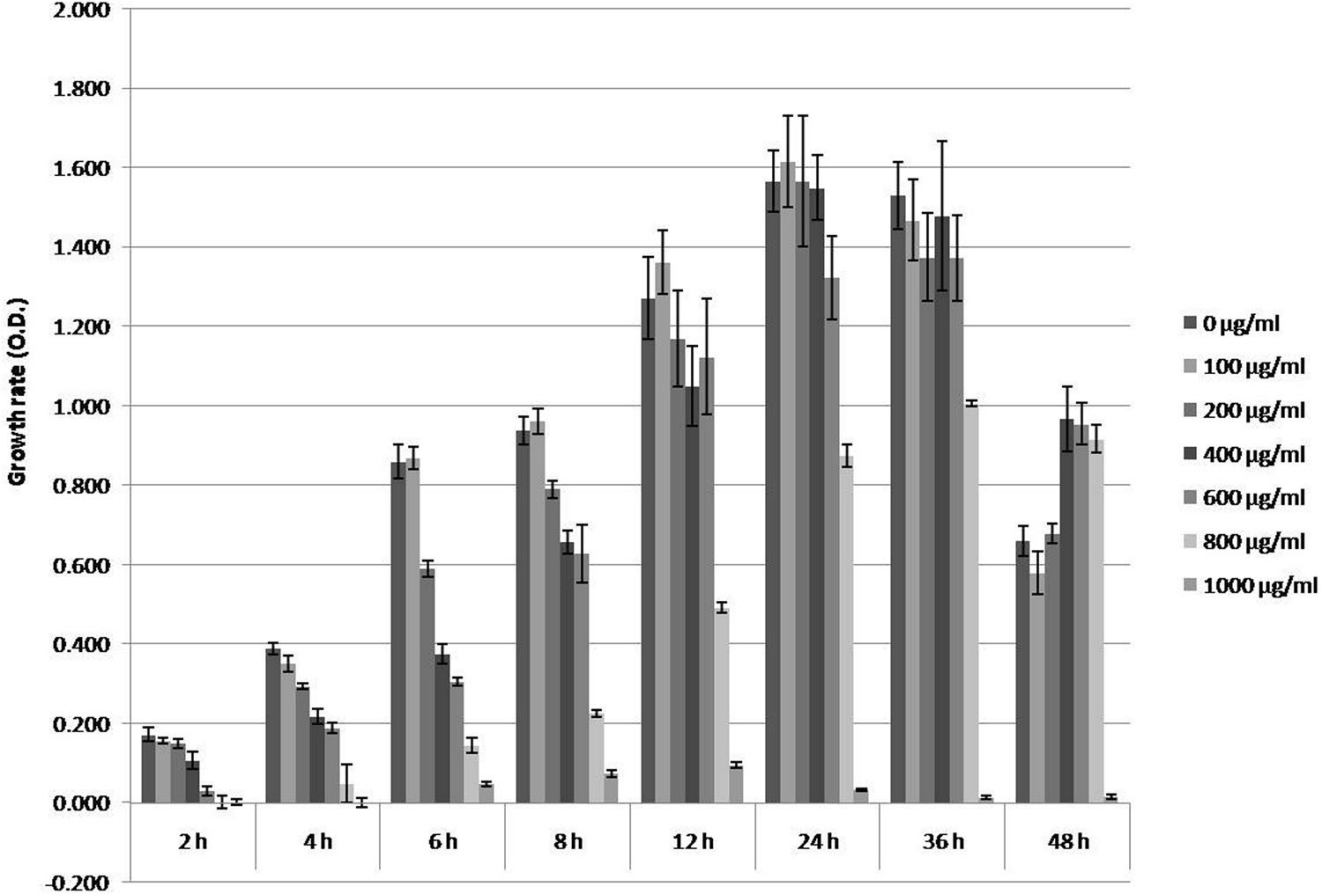
Rate of CLA production by highest CLA-producing *B. fibrisolvens* VIII strain (Isolate VIII)

CLA production potential of 31 pure *Butyrivibrio* strains was analysed with different concentration of free LA (0, 200, 400, 600, 800 μg/ml) at different time interval (0, 2, 4, 6, 12, and 24 h), and highest CLA production was found in case of strain 4a which produced 140.77 μg/ml when incubated with 200 μg/ml LA at 2 h of incubation in DSMZ704 medium, converting almost 70 % of total LA into CLA (Fig. 4). Among 31 pure strains of *Butyrivibrio*, 4 strains converted more than 50 % LA to CLA at various concentration of LA supplementation and at different time intervals. Furthermore, it was also found that 10 strains (32.25 %) produced highest CLA when incubated with 200 μg/ml of LA, followed by 7 strains (22.58 %) with 400 μg/ml LA, 8 strains (25.8 %) with 600 μg/ml LA and 6 strains (19.35 %) when incubated with 800 μg/ml LA.

**Fig. 5:**
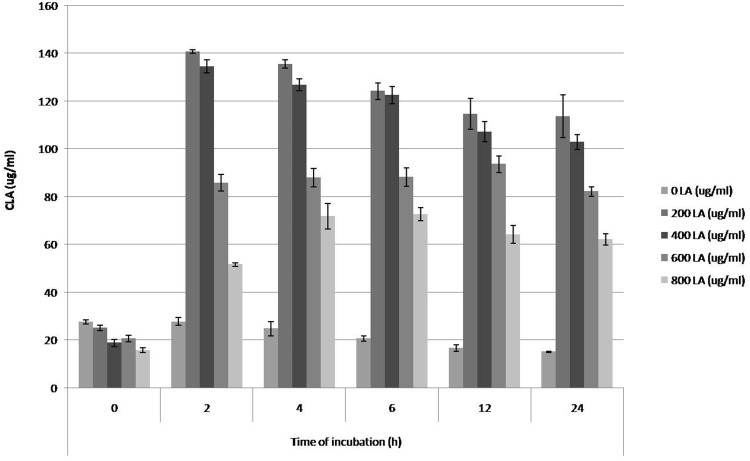
Growth rate of *B. fibrisolvens* VIII (Isolate VIII) incubated in different concentrations of LA for 48 h

The highest CLA-producing strain, *B. fibrisolvens* 4a was further selected to investigate the effect of free LA on the growth of bacterium. Biohydrogenation kinetics of *B. fibrisolvens* 4a with different LA concentrations (0, 100, 200, 400, 600, 800, and 1000 μg/ml) for 48 h cultivation period (0, 2, 4, 6, 8, 12, 24, 36, and 48 h) was observed. Steady growth was evident up to 24 h of cultivation period with 100–200 μg/ml free and pure LA. Further increase in cultivation period as well as LA concentration resulted in lower growth pattern (Fig. 5; Table 3). However, a significant decline was observed when the isolate was cultivated with medium having LA concentration greater than 600 μg/ml LA. Also after 36 h of cultivation period in medium with different LA concentrations, the growth was drastically decreased uniformly.

**Table 3:** In vitro CLA production by *Butyrivibrio* isolates showing different response of strains from similar species when incubated with linoleic Acid (LA)

LA concentration (ug/ml)	CLA production (ug/ml) by *Butyrivibrio* strains (I–XVI)															
	I	II	III	IV	V	VI	VII	VIII (4a)	IX	X	XI	XII	XIII	XIV	XV	XVI
0	13.53	18.73	16.98	15.41	18.45	19.31	20.41	26.27	21.69	13.79	17.84	15.73	18.14	17.55	17.39	18.31
200	69.80	81.41	72.99	88.82	84.72	91.03	80.62	**140.77**	90.15	57.99	81.00	70.30	91.74	82.46	80.73	93.65
400	75.35	67.45	61.10	81.82	98.88	96.29	79.88	134.11	89.84	59.35	77.57	66.20	88.54	91.60	78.16	99.09
600	66.92	73.82	62.97	71.26	123.51	93.73	106.49	85.55	84.39	62.47	71.99	56.38	71.63	75.14	69.17	87.36
800	67.49	62.95	60.09	56.86	92.91	84.60	70.55	50.91	67.99	63.75	54.67	82.27	72.12	105.78	91.71	77.40

LA concentration (ug/ml)	CLA production (ug/ml) by Butyrivibrio strains (XVII–XXXI)														
	XVII	XVIII	XIX	XX	XXI	XXII	XXIII	XXIV	XXV	XXVI	XXVII	XXVIII	XXIX	XXX	XXXI
0	14.08	17.99	11.76	14.94	16.12	12.41	14.13	26.09	17.82	10.49	16.68	12.45	14.06	13.06	10.10
200	70.82	70.05	52.14	69.24	49.17	105.18	65.45	69.76	77.46	36.24	30.53	43.61	61.15	45.20	43.90
400	69.93	83.90	50.45	83.40	49.74	65.58	72.50	113.43	75.98	26.18	32.83	47.70	32.75	50.76	41.61
600	66.40	86.82	60.87	67.65	57.33	58.05	116.60	107.08	67.65	38.04	40.58	51.20	34.24	44.58	48.60
800	85.42	68.80	50.65	59.38	38.30	51.32	60.98	90.25	68.45	46.68	46.50	44.67	29.68	37.81	43.61

Bold value indicates highest CLA production by *B. fibrisolvens* strain at 200 ug/ml LA concentration

## Discussion

CLA, a by-product of rumen biohydrogenation, has several proven health-promoting effects and thus has been a subject of constant study in the recent years. To sufficiently access its health effects, it is necessary to have its constant optimal intake as large doses of synthetic CLA supplements have been shown to be having adverse effects (Ip et al. 1997). Natural CLA is produced in rumen as a result of biohydrogenation with interventions from rumen bacterial population. Apart from *Butyrivibrio* spp. several other bacterial species such as *Lactobacillus acidophilus* (Ogawa et al. 2001; Alonso et al. 2003; Yadav et al. 2007), *Bifidobacterium* spp., particularly *B. breve*, *B. dentium*, *B. longum* (Coakley et al. 2003) and various strains of lactic acid bacteria (Ogawa et al. 2005) have been shown to produce CLA. Among 15 isomers of CLA, ruminal bacteria have been reported to produce significant amounts of the *cis*-9, *trans*-11 and *trans*-10, *cis*-12 isomers only (Griinari and Bauman 1999). Microbial CLA production, in addition to rumen bacteria, has been reported for *Propionibacteria*, particularly *Propionibacterium freudenreichii*, used as dairy starter cultures (Jiang et al. 1998). Kishino et al. (2002) found that *Lactobacillus plantarum* (*L. Plantarum*) formed high levels of CLA from free LA upon extended incubation. Amongst all rumen bacteria *B. fibrisolvens* has been shown to be having high ability to isomerise LA to CLA (Kim et al. 2000; Kim 2003). Several strains having high CLA production capability have been identified as potential probiotics (Fukuda et al. 2006).

Studies have also demonstrated abundance of *Butyrivibro spp.* in rumen; however, no attempt has been made to extend the knowledge of *Butyrivibrio* spp. diversity in Indian origin ruminants. Present study was undertaken to assess the CLA production potential of *Butyrivibrio* spp. isolated from cattle of Indian origin and to assess diversity among this species in the ruminants.

Research findings have indicated that *Butyrivibrio* spp. is amongst some of the major culturable occupants of rumen. At least 10–30 % of rumen microbiota is suggested to be composed of this CLA-producing species (Kopeeny et al. 2003; Jarvis and Moore 2010; Zhu et al. 2014). High intra-species diversity is a characteristic feature of *Butyrivibrio* spp (Kopecny et al. 2003). In the present study also the isolated population included *B. fibrisolvens*, *B. proteoclasticus* and *B. hungatei*. It could also be noted that bacteria of unknown species were also identified during the course of present study which can also play, directly or indirectly, a vital role in biohydrogenation process. Decrease in C:18 concentration has earlier been found to be associated with disappearance of a particular bacterial species such as *Lachnospiraceae s*trains (Boeckaert et al. 2008).

 The production of CLA by *Butyrivibrio* spp. displayed substantial interspecies variation. Among all the strain tested, *B. fibrisolvens* was the most efficient CLA producer (Kepler et al. 1966; Kim et al. 2000; Kim 2003). It is evident from the observations recorded during the course of experiment that CLA production ability does not follow a single pattern and varies with time of incubation and also with concentration of free LA supplemented; establishing that CLA production potential is highly a strain-specific attribute (Table 3). The ability of different strains to isomerise LA to CLA has been demonstrated to vary corresponding to CLA isomerase (CLA-I) and CLA reductase (CLA-R) activity of a strain. CLA was found to be accumulating only after the TH1 culture growth ceased in a study by Fukuda et al. (2005) owing to high CLA-R activity. In the present study also most of the strains showed decrease in CLA concentration after a certain cultivation period.

LA being the precursor in biohydrogenation process affects the products, whether intermediates such as CLA or final products such as VA and SA (Lourenco et al. 2010). Polan et al. (1964) also observed high accumulation of CLA and trans-18:1 with the increase concentration of LA in the culture media. However, CLA production with respect to cultivation period has been observed to be highly strain specific. In some cases, as in a study by McKain et al. (2004) supplementation of LA resulted in increase of lag period and thus low CLA production was recorded in the first 8 h. High CLA production was recorded during 8–18 h of incubation (log phase) because activity of growing cells was higher for biohydrogenation than stationary phase (McKain et al. 2004). In the present study, maximum CLA production was recorded at 2 h of incubation, i.e. in the lag phase itself. High metabolic functionality and thus high metabolite production in lag phase is not a new concept. It has been shown that some of the microbial species have high metabolite production capability in the lag phase of their growth. However, studies are more clear in case of eukaryotes where significant upregulation in expression of genes pertaining to translation, protein folding, modification, translocation and degradation, ribosome biogenesis, transcription, RNA processing, cell polarity, cell division, and cell cycle control have been reported (Brejning et al. 2003, 2005). Rolfe et al. (2011) have indicated that lag phase is associated with high uptake of metals and minerals during cultivation of Salmonella. Additionally, some of the enzymes are reported to be produced in their most active forms in lag phase of bacterial growth (Ponce et al. 1995). An upregulated expression of genes responsible for the involved enzymes might well be the reason of high CLA production within 2 h of cultivation period in our experiments. Also the bacterial isolates were in highly active state consequent to repeated sub-culturing prior to actual inoculation in experimental medium, which might have reduced their lag phase. Moreover, as we know now that lag phase is an important transient plateau before the start of exponential phase but is still scarcely understood. A more detailed study at transcriptome level can provide important leads into the responsible reasons regarding CLA production.

It was observed that CLA produced by bacterial strain 4a, *B. fibrisolvens* VIII (highest CLA-producing strain) was almost 3.5 times higher than the CLA produced by bacterial strain XXVII *B. proteoclasticus* 20 (lowest CLA-producing strain). In the present study, *B. fibrisolvens* VIII strain produced maximum CLA during the first 2 h of incubation and at 200 μg/ml LA supplemented to the growth media and it was gradually decreased further with time and LA concentration. It may be that more CLA is being produced with time but at the same time, the CLA formed is getting converted to VA. Studies indicate that *B. fibrisolvens*, a representative PUFA-hydrogenating ruminal bacterium, produces the highest levels of t-VA from LA among bacteria surveyed (Harfoot and Hazlewood 1997; Jiang et al. 1998; Kim et al. 2000) and can be found in the digestive tract of many animals including human bowel (Barcenilla et al. 2000). It is known that *B. fibrisolvens* isomerizes LA to c9, t11-CLA via LA isomerase (LA-I), and then reduces to t-VA via CLA reductase (CLA-R) (Kepler et al. 1966).

Growth of the bacteria was inhibited in presence of PUFA and it was inversely proportional to the LA concentration. The polyunsaturated fatty acids (PUFA) are toxic to *B. fibrisolvens* as they disrupt the lipid bilayer (Keweloh and Heipieper 1996) or because it takes longer time to bio-hydrogenate the whole molecule (Li et al. 2012). Average growth of the isolated bacteria which was lower with increasing LA supplementation indicated that the toxicity of unsaturated fatty acid depends on the number of double bonds, i.e. EPA > DHA > LNA > LA (Maia et al. 2007).

 In conclusion, the present study describes the diversity and CLA-producing ability of *Butyrivibrio* from Indian ruminants; thus leading to the identification of *B. fibrisolvens* VIII strain as the most efficient CLA producer, among the screened rumen microbiota. Furthermore, a range of other rumen microbes exhibiting CLA-producing capabilities was also identified. Considering the health beneficial properties of CLA, it is captivating to discover probiotic *Butyrivibrio* strains with high ability to produce CLA to use them as feed additive for ruminants. This was a preliminary study to isolate high CLA-producing bacterial strains; further research work is required to ascertain the use of such strains as potential probiotics for animals.

## References

[CR1] Alonso L, Cuesta EP, Gilliand SE (2003). Production of free conjugated linoleic acid by *Lactobacillus acidophilus* and *Lactobacillus casei* of human intestinal origin. J Dairy Sci.

[CR2] Altschul SF, Gish W, Miller W, Myers FW, Lipman DJ (1990). Basic local alignment search tool. J Mol Biol.

[CR3] Barcenilla A, Pryde SE, Martin JC, Duncan SH, Stewart CS, Henderson C, Flint HJ (2000). Phylogenetic relationships of butyrate-producing bacteria from the human gut. Appl Environ Microbiol.

[CR4] Barrett E, Ross RP, Fitzgerald GF, Stanton C (2007). Rapid screening method for analyzing the conjugated linoleic acid production capabilities of bacterial cultures. Appl Environ Microbiol.

[CR5] Boeckaert C, Vlaeminck B, Fievez V, Maignien L, Dijkstra J, Boon N (2008). Accumulation of trans C18:1 fatty acids in the rumen after dietary algal supplementation is associated with changes in the *Butyrivibrio* community. Appl Environ Microbiol.

[CR6] Brejning J, Jespersen L, Arneborg N (2003). Genome-wide transcriptional changes during the lag phase of *Saccharomyces cerevisiae*. Arch Microbiol.

[CR7] Brejning J, Arneborg N, Jespersen L (2005). Identification of genes and proteins induced during the lag and early exponential phase of lager brewing yeasts. J Appl Microbiol.

[CR8] Chinnadurai K, Kanwal HK, Tyagi AK, Stanton C, Ross P (2013). High conjugated linoleic acid enriched ghee (clarified butter) increases the antioxidant and antiatherogenic potency in female Wistar rats. Lipids Health Dis.

[CR9] Coakley M, Ross RP, Nordgren M, Fitzgerald G, Devery R, Stanton C (2003). Conjugated linoleic acid biosynthesis by human-derived *Bifidobacterium* species. J Appl Microbiol.

[CR10] Dilzer A, Park Y (2015). Implication of conjugated linoleic acid (CLA) in human health. Crit Rev Food Sci Nutr.

[CR11] DMSZ, 1993.DMSZ 704 medium. http://www.dmsz.de/media/med704.html

[CR12] Fukuda S, Furuya H, Suzuki Y, Asanuma N, Hino T (2005). A new strain of *Butyrivibrio fibrisolvens* that has high ability to isomerize linoleic acid to conjugated linoleic acid. J Gen Appl Microbiol.

[CR13] Fukuda S, Suzuki Y, Murai M, Asanuma N, Hino T (2006). Isolation of a novel strain of *Butyrivibrio**fibrisolvens* that isomerizes linoleic acid to conjugated linoleic acid without hydrogenation, and its utilization as a probiotic for animals. J Appl Microbiol.

[CR14] Griinari JM, Bauman DE (1999) Biosynthesis of Conjugated Linoleic Acid and its composition, incorporation into meat and milk in ruminants. In: Advances in CLA research. AOCS Press, Champaign, Il:180–200

[CR15] Ha YL, Grimm N, Pariza MW (1989). Newly recognized anticarcinogenic fatty acids: identification and quantification in natural and processed cheeses. J Agric Food Chem.

[CR16] Hall TA (1999). BioEdit: a user-friendly biological sequence alignment editor and analysis program for Windows 95/98/NT. Nucl Acids Symposium Series.

[CR17] Harfoot CG, Hazlewood GP (1997) Lipid Metabolism in the Rumen. In: Hobson PN, Stewart 222 DS (ed) The Rumen Microbial Ecosystem, Chapman & Hall, London, pp 382–426

[CR18] Hino T, Suzuki K, Ohkawara S, Miwa T, Asanuma N (2012). Effects of oral administration of *Butyrivibrio**fibrisolvens* MDT-1 on the development and healing of atopic dermatitis in NC/Nga mice. Eur J Dermatol.

[CR19] Holt JG, Krieg NR, Sneath PHA, Staley JT, Williams ST (1994). Bergey’s manual of determinative bacteriology.

[CR20] Ip C, Jiang C, Thompson HJ, Scimeca JA (1997). Retention of conjugated linoleic acid in the mammary gland is associated with tumor inhibition during the postinitiation phase of carcinogenesis. Carcinogenesis.

[CR21] Jarvis GN, Moore ERB, Timmis KN (2010). Lipid metabolism and the rumen microbial ecosystem. Handbook of hydrocarbon and lipid microbiology.

[CR22] Jiang J, Bjorck L, Fonden R (1998). Production of conjugated linoleic acid by dairy starter cultures. J ApplMicrobiol.

[CR23] Kepler CR, Hirons KP, McNeil JJ, Tove SB (1966). Intermediates and products of the biohydrogenation of linoleic acid by Butyrivibrio fibrisolvens. J BiolChem.

[CR24] Keweloh H, Heipieper HJ (1996). Trans unsaturated fatty acids in bacteria. Lipids.

[CR25] Kim YJ (2003). Partial inhibition of biohydrogenation of linoleic acid can increase the conjugated linoleic acid production of *Butyrivibrio fibrisolvens* A38. J Agri Food Chem.

[CR26] Kim YJ, Liu RH, Bond DR, Russell JB (2000). Effect of linoleic acid concentration on conjugated linoleic acid production by *Butyrivibrio fibrisolvens* A38. Appl Environ Microbiol.

[CR27] Kishino S, Ogawa J, Omura Y, Matsumura K, Shimizu S (2002). Conjugated linoleic acid production from linoleic acid by lactic acid bacteria. J American Oil Chem Soc.

[CR28] Kopeeny J, Zorec M, Mrazek J, Kobayashi Y, Marinsek-Logar R (2003). *Butyrivibrio**hungatei* sp. nov. and *Pseudobutyrivibrio**xylanivorans* sp. nov., butyrate-producing bacteria from the rumen. Int J Syst Evol Microbiol.

[CR29] Kritchevsky D (2000). Antimutagenic and some other effects of conjugated linoleic acid. British J Nutr.

[CR30] Li D, Wang JQ, Bu DP (2012). Ruminal microbe of biohydrogenation of trans-vaccenic acid to stearic acid in vitro. BMC Res Notes.

[CR31] Lin TY, Lin CW, Lee CH (1999). Conjugated linoleic acid concentration as affected by lactic cultures and added linoleic acid. Food Chem.

[CR32] Lourenco M, Ramos-Morales E, Wallace RJ (2010). The role of microbes in rumen lipolysis and biohydrogenation and their manipulation. Animal.

[CR33] Maia MRG, Chaudhary LC, Figueres L, Wallace RJ (2007). Metabolism of polyunsaturated fatty acids and their toxicity to the microflora of the rumen. Antonie Van Leeuwenhoek.

[CR35] McKain N, Chaudhary LC, Walker ND, Pizette F, Koppova I, McEwan NR, Kopecny J, Vercoe PE, Wallace RJ (2004) Relation between phylogenetic position and fatty acid metabolism of different Butyrivibrio isolates from the rumen. Repr Nutr Develop 44:64 10.1007/s10482-006-9121-7 17077990

[CR36] Ogawa J, Matsumura K, Kishino S, Omura Y, Shimizu S (2001). Conjugated linoleic acid accumulation via 10-hydroxy-12-octadecaenoic acid during microaerobic transformation of linoleic acid by *Lactobacillus acidophilus*. Appl Environ Microbiol.

[CR37] Ogawa J, Kishino S, Ando A, Sugimoto S, Nishara K, Shimizu S (2005). Production of conjugated linoleic acid by lactic acid bacteria. J Biosci Bioengg.

[CR38] Ohkawara S, Furuya H, Nagashima K, Asanuma N, Hino T (2006). Effect of Oral Administration of *Butyrivibrio fibrisolvens* MDT-1 on Experimental Enterocolitis in Mice. Clin Vaccine Immunol.

[CR39] Polan CE, McNeill JJ, Tove SB (1964). Biohydrogenation of unsaturated fatty acids by rumen bacteria. J Bacteriol.

[CR40] Ponce E, Flores N, Martinez A, Valle F, Bolívar F (1995). Cloning of the two pyruvate kinase isoenzyme structural genes from *Escherichia coli*: the relative roles of these enzymes in pyruvate biosynthesis. J Bacteriol.

[CR34] Rolfe MD, Rice CJ, Lucchini S, Pin C, Thompson A, Cameron AD, Alston M, Stringer MF, Betts RP, Baranyi J, Peck MW, Hinton JC (2011). Lag phase is a distinct growth phase that prepares bacteria for exponential growth and involves transient metal accumulation. J Bacteriol.

[CR41] Rosberg-Cody E, Ross RP, Hussey S, Ryan CA, Murphy BP, Fitzgerald GF, Devery R, Stanton C (2004). Mining the microbiota of the neonatal gastrointestinal tract for conjugated linoleic acid-producing bifidobacteria. Appl Environ Microbiol.

[CR42] Willems A, Amat-Marco M, Collins MD (1986). Phylogenetic analysis of *Butyrivibrio* strains reveals three distinct groups of species within the *Clostridium subphylum* of the gram-positive bacteria. Intl J Syst Bacterial.

[CR43] Yadav H, Jain S, Sinha PR (2007). Production of free fatty acids and conjugated linoleic acid in probiotic dahi containing *Lactobacillus acidophilus* and *Lactobacillus casei* during fermentation and storage. Int Dairy J.

[CR44] Zhu Z, Hang S, Mao S, Zhu W (2014). Diversity of *Butyrivibrio* Group bacteria in the rumen of goats and its response to the supplementation of garlic oil. Asian Australas. J Anim Sci.

